# Toward a More Sustainable Trajectory for E-Waste Policy: A Review of a Decade of E-Waste Research in Accra, Ghana

**DOI:** 10.3390/ijerph14020135

**Published:** 2017-01-29

**Authors:** Kurt Daum, Justin Stoler, Richard J. Grant

**Affiliations:** 1 Department of Geography and Regional Studies, University of Miami, Coral Gables, FL 33146, USA; k.daum@umiami.edu (K.D.); rgrant@miami.edu (R.J.G.); 2 Department of Public Health Sciences, Miller School of Medicine, University of Miami, Miami, FL 33136, USA; 3 Abess Center for Ecosystem Science and Policy, University of Miami, Coral Gables, FL 33146, USA; 4 Department of Geography, Environment Management, and Energy Studies, University of Johannesburg, Johannesburg 2006, South Africa

**Keywords:** e-waste, recycling, governance, environmental health, public policy, Ghana

## Abstract

Global flows of e-waste from the Global North to the Global South continue to damage local environments and harm human health. Weak e-waste regulations and limited use of safety measures for e-waste workers in Accra, Ghana, foster an exploitative environment within the industry, and pose health risks for those working and living near e-waste processing sites. This paper presents an integrated review of over 40 e-waste studies specific to Accra, with particular emphasis on the well-studied e-waste processing site in Agbogbloshie, and synthesizes the existing research base across interdisciplinary themes of human health, environmental health, globalization, trade and informalization, and public policy. Despite significant international attention to Accra’s e-waste problem, loopholes within international environmental regulations and treaties provide few incentives and resources for Ghana to strengthen protections for human and environmental health. After a decade of e-waste research in Accra, the crisis continues to intensify; we present a renewed vision for sustainable e-waste policy reform in Ghana and beyond.

## 1. Introduction

Many cities in Africa have become receptacles for the Global North’s discarded electronic waste (e-waste), and the industry that has emerged around e-waste disassembly is causing environmental catastrophes. Agbogbloshie, a slum in the heart of Accra, Ghana, has achieved notoriety as one of the most polluted slums in the world. The Blacksmith Institute (renamed Pure Earth in 2015) rated the Agbogbloshie e-waste site as “among the top ten most toxic sites in the world” [1]. However, the empirical basis for this claim is far from clear (based on a comparison of findings from sampled sites and populations from research funded by the organization, as well as from the secondary literature) as are reoccurring, alarming curated media reporting that Agbogbloshie is the largest digital dumpsite on the planet [2,3,4]. We know that Agbogbloshie is generally not comparable to Chinese sites such as Guiyu, but the international notoriety of the Accra site and the accumulation of knowledge about this e-waste hub make Accra a compelling case study. 

The municipal authority, the Accra Metropolitan Assembly (AMA), is acutely aware of elevated environmental health risks and hazards in this site but they have been reluctant to tackle the problem. Ghana’s e-waste activities generate US$105–268 million annually and sustain the livelihoods of at least 200,000 people nationwide [5]. The Agbogbloshie site alone provides livelihood opportunities of various sorts to approximately 4500–6000 workers and perhaps another 1500 indirectly [6]. In June 2015, just a few weeks after a disastrous flood that killed approximately 200 Accra citizens, the AMA conducted a demolition (i.e., bulldozing) exercise in Old Fadama, a large urban slum adjacent to Agbogbloshie, under the guise of relieving encroachment around the Korle Lagoon and freeing the flow of water. The causes of the flood were undoubtedly multi-faceted and related to climate change, urban sprawl, weak storm infrastructure, and inadequate water management, and certainly not solely due to e-waste accumulations. However, the popular narrative about a vast e-waste site with e-waste debris piling up was an important claim. Hundreds of shacks and structures along the Korle Lagoon were demolished, resulting in the forced eviction of thousands of community residents. The clearing exercise included some e-waste processing sites, but the AMA spared the vast majority of the e-waste processing hub after a series of spirited—and occasionally violent—demonstrations by local residents. The AMA allegedly had opted to limit the bulldozing to 50 m from the lagoon rather than the 100-m zone previously stipulated by municipal authorities in earlier dialogues with the local community, which would have razed larger parts of the e-waste processing site. However, some bulldozing occurred at distances greater than 100 m from the lagoon, and fear of a more extreme backlash from the cadre of informal e-waste workers discouraged the government from bulldozing even further into the community. Despite the absence of additional bulldozing operations in 2016, the municipal authority has given notice of future demolitions.

Various scholars have researched this site extensively, and the media have documented most of the visible aspects of e-waste processing (burning, air pollution, marginal livelihoods, and child labor). The collective body of e-waste research literature pertaining to e-waste in Ghana is quite vast but tends to focus on human and environmental health, globalization, trade and informalization, and policy. It is clear that airborne chemicals, most notably polybrominated diphenyl ethers (PBDEs), are leaching into bodily tissues of workers and innocent civilians, as well as into the air, soil, and water of nearby communities. Research into the intangible quality of life of those involved in the e-waste industry, as well as into tactical governance and policy strategies for protecting it, is far more limited. To date no comprehensive synthesis of the African e-waste literature has been undertaken, and thus many study results are weakly integrated. We contend that e-waste is a multidimensional phenomenon and that local governments would benefit from a comprehensive synthesis of the scholarly literature in order to better guide e-waste policy along a more sustainable trajectory. Such a trajectory must take into consideration the extensive damage that has already been done so that demolitions and “out of sight, out of mind” cease to prevail as the most expedient reaction. 

Previous reviews of the e-waste literature have tended to take a global focus of e-waste flows and governance issues [7,8]. Our paper positions Accra, Ghana, as a case study for synthesizing what we have learned after over a decade of interdisciplinary e-waste research. We begin by reviewing the Accra-focused e-waste literature pertaining to human and environmental health to highlight the evolution and proliferation of e-waste’s damaging effects. We expand on this by examining the e-waste management system in Accra by analyzing the legislation and institutional hierarchies in place, and then summarize the e-waste trade’s social implications and discuss potential solutions to the crisis. We present an integrated picture of the Ghanaian e-waste experience through a holistic examination of the e-waste literature pertaining to the case study of Agbogbloshie. We aspire to better understand the realities of the global e-waste dilemma and identify opportunities where we can mitigate the numerous externalities of the e-waste industry, especially as it manifests in the daily lives of residents in African cities, and ultimately move the e-waste discussion toward a more sustainable pathway. 

## 2. Methods

### 2.1. Literature Review

This paper is drawn from an interdisciplinary body of scholarly literature pertaining to e-waste, with focus on the Ghana experience, and contextualized by the global e-waste literature and local media and non-governmental organization (NGO) reports. We reviewed all available Ghana-related e-waste scholarly literature published in English before March 2016 that was available through major research databases such as PUBMED, SCOPUS, and ScienceDirect. The Ghana-specific e-waste literature dated back to 2002 and included over 40 sources as of 2016. We used a grounded theory approach to evaluate the main content of each article and derive core themes that were the basis for synthesizing the literature. These emergent themes comprise the organizational framework for the rest of this article: human health impacts, environmental health impacts, the e-waste management system, the e-waste worker experience, and policy considerations. 

### 2.2. Agbogbloshie: Social and Geographic Context

**Figure 1:**
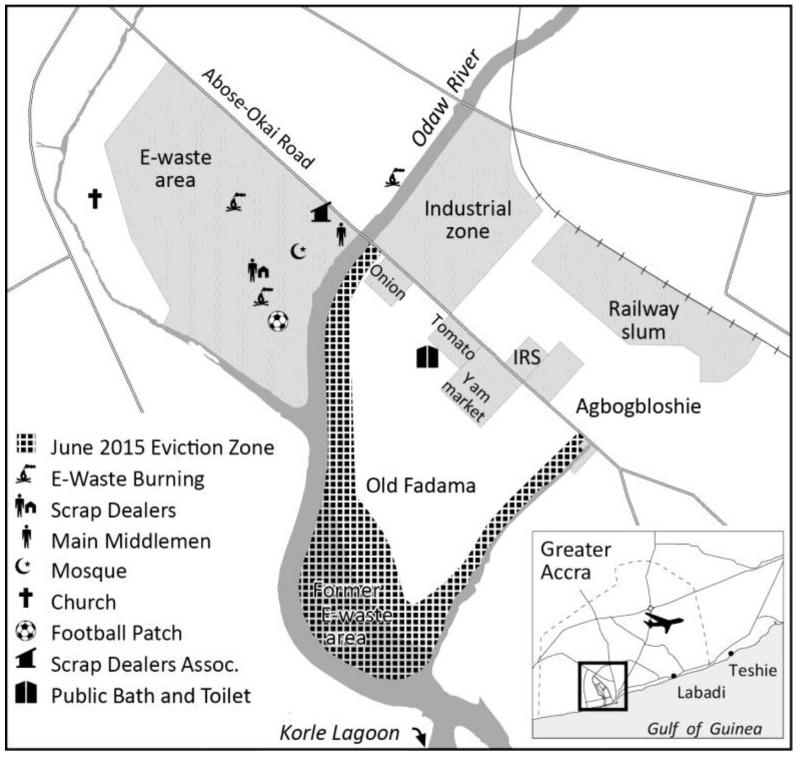
Urban features related to the e-waste processing industry within the Agbogbloshie and Old Fadama neighborhoods.

Agbogbloshie is an old neighborhood in Central Accra that has become an internationally known hotspot of e-waste recycling [6]. A large informal settlement, Old Fadama, lies adjacent to Agbogbloshie, just a few hundred meters southeast of the central waste dump where a considerable portion of recycling practices occur (Figure 1). While Agbogbloshie and Old Fadama are technically separated by Abose-Okai Road, they function as an extended community (the names are often used imprecisely and interchangeably) and together comprise one of Ghana’s largest urban slums. Early settlers arrived to this area in 1981; it has since attracted economic migrants from various parts of the country (typically northern Ghana) who seek employment. Another of Old Fadama’s pull factors is its low cost of living; the neighborhood has some of the cheapest rents in the city [9]. Based on a 2009 community enumeration, the roughly 0.4 km^2^ area was home to 79,684 individuals [10]. Despite having little to do with the generation of e-waste, these low-income inhabitants live the closest to the city’s e-waste hub due the ecological distribution of Accra’s general waste management system [11], which doubly marginalizes residents of Agbogbloshie’s informal settlements. 

The e-waste site is integrated within the social geography of the community. Within the scrap processing site southeast of the main waste dump (Figure 1) are a mosque and an informal football patch where workers—both adults and children—play amid scraps of e-waste. Other places of worship include a church southwest of the plastic processing site and another mosque northwest of the same site. A church-owned basketball court located nearly 140 m southwest of the plastic processing site offers another space for workers of all ages to play during their leisure time. Lastly, a goat pasture covered in e-waste is located southwest of the scrap processing site and main waste dump. Livestock graze on the soiled land [3,12]. Due to spatial proximity, all of these areas within the greater Agbogbloshie community are subject to airborne exposure from e-waste byproducts. 

Local importers, including formal business importers and informal small-scale importers, bring electronics and electronic equipment (EEE) into Ghana [12]. Formal importers negotiate import deals with manufacturers and distributers from the developed world, mainly from Europe; when negotiated this way, EEE in working condition comprise roughly 70% of shipments. Informal importers usually reside abroad and import shipments of EEE intermittently. EEE quality generally decreases with informal importers to 60% working condition units per shipment. More recently local operators note that the percentage of non-working devices has risen in parallel with the expansion of the processing of valuable fractions (e.g., copper) from e-waste and the growth of scrap exports [13]. It is important to emphasize that e-waste is only part of the economic geography of Agbogbloshie. Many informal workers and families also reside there, and many residents work in other parts of Accra. Agbogbloshie has a well-developed truck repair cluster, food markets, and basic retailing. Importantly, this e-waste hub has long been integrated with secondary e-waste processing sites. In particular, since June 2015 e-waste burning has shifted to nearby urban areas (outside of Accra) such as Ashiaman and Pantang. 

## 3. Human Health Impacts

Airborne, waterborne, foodborne, and dust-borne toxins originating from the Agbogbloshie dumpsite are the main source of contamination in human tissue and bodily fluids. With little-to-no protective measures in use, e-waste workers participate in recycling activities that endanger not only themselves, but also local inhabitants, particularly children and infants. Due to the pervasive nature of environmental toxins in the local atmosphere, residents of the nearby settlement of Old Fadama and those working and residing in the central business district (CBD) are at risk of experiencing high exposure levels on a daily basis. As the e-waste crisis becomes increasingly publicized, researchers are paying closer attention to the effects of environmental pollutants on the human body; as of spring 2016, little is known about the long-term effects of repeat exposure or the spatial reach of these locally-generated hazards.

### 3.1. Toxins Found in E-Waste Workers

Blood samples of e-waste workers have been shown to exhibit elevated concentrations of heavy metals and flame retardants [14,15]. For the exposed population, sampled mean serum levels of cobalt, chromium, copper, iron, selenium, and zinc were significantly higher compared to a control group [14]. Of the polychlorinated dibenzo-dioxins/furans (PCDD/Fs) found in blood samples, 1,2,3,7,8-PentaCDD, 1,2,3,7,8,9-HexaCDD, 1,2,3,4,6,7,8-HexaCDD, and OctaCDD concentrations are significantly more elevated in exposed e-waste workers than in subjects with no exposure [15].

Concentrations of heavy metals and polycyclic aromatic hydrocarbons (PAHs), which are general byproducts of incomplete burning and known carcinogens, have been detected in urine samples of Abgogbloshie e-waste workers [14,16,17]. Concentrations of iron, tin, lead, barium, manganese, and zinc have also been shown to be much higher among e-waste workers [14,16]. Five different PAH metabolites were detected in worker urine samples; these findings were not linked to any pathological findings, but pulmonary issues were common with about two thirds of exposed individuals reporting a cough and about one quarter reporting chest pains [17]. 

Breast milk samples from women residing near the Agbogbloshie Market contained abnormally high concentrations of polychlorinated biphenyls and other brominated flame retardants like PBDEs and hexabromocyclododecanes (HBCDs). A sample of mothers had mixed diets that included various meats and fish, the latter often purchased nearby. It was revealed that mothers in Accra had more PBDEs than mothers in Tamale, a secondary e-waste site [18], spurring fears that maternal health may be declining at a faster rate in and around Agbogbloshie due to the intensity of recycling activities and urban pollution.

### 3.2. Neonatal Health

The health risks for fetuses and infants in Agbogbloshie are higher than those of adults because neonates and children are only beginning their bodily developmental processes. Indeed, heavy metals and chemical compounds found within electronic devices have been linked to neurodevelopmental disorders and/or fetal perturbations [19,20]. Lead, mercury, and cadmium exposure as well as PBDE, polychlorinated biphenyl (PCB), PCDD/F, and PAH exposure are all linked to negative cognitive development effects in children maturing in e-waste neighborhoods; these toxins can generally be found in the air and dust among other environmental mediums [21]. High blood lead levels have also been correlated with negative effects on mood, physical activity, and adaptability, while also affecting bone resorption and general physical development over time [19,22].

Infants are highly susceptible to metal and chemical exposure in Agbogbloshie because of the abundance of exposure routes. While adults endure persistent exposure to e-waste toxins through air, dust, water, and food, nursing infants face an additional potential exposure via breast milk. HBCDs and PBDEs found in breast-feeding mothers have been transmitted to their children in Agbogbloshie [18]. Daily feeding and repetitive exposures heightens the level of risk, with mounting and unknown interactions on cognitive deficiencies as the infant matures.

## 4. Environmental Health Impacts

Rudimentary recycling techniques practiced by informal e-waste processers in Accra exacerbate the release of environmental toxins that pollute and contaminate landscapes, waters, and biota of Agbogbloshie. The hazardous environmental health effects are due to the survivalist functioning of an informal and small-scale recycling industry that is starved of capital and operates without oversight. The Agbogbloshie informal settlement, e-waste hub, and food markets have all grown in size and density over the last decade (until the demolition of June 2015 when the AMA destroyed several hundred informal structures), leaving the vicinity peppered with remnants of old electronic goods, dust, and ashes—as well as smashed timber and metals from slum demolitions. Waste left in fields and nearby bodies of water is ingested by animals and marine life, thus creating entry points for toxins into non-human ecological systems. High residential density and e-waste processing in very close proximity to food markets only heightens the degree of exposure for human-environmental systems.

### 4.1. Toxins Found in Air, Soil, Dust, and Ash

Air samples from the Agbogbloshie Market have revealed heavy metals and polychlorinated naphthalene (PCN) congeners [23,24]. Workers’ physical breathing spaces contained elevated concentrations of aluminum, copper, and iron, while lead concentrations were four times higher than the allowable United States Environmental Protection Agency (USEPA) ambient air quality [23]. PCNs, which are industrial organic contaminants, were much more prevalent in Accra than they were in other Ghanaian sites due to the amount of e-waste cable burning that takes place [24].

E-waste related activities occurring in the various processing and waste sites in Agbogbloshie have caused metals to leach into the area’s soils and to create layers of ash and dust [23,25,26,27]. Over half of 100 soil samples collected from dumping and processing sites contained lead concentrations that exceeded USEPA lead standards [23]. In ash samples, concentrations of copper, zinc, lead, and tin were extremely high (up to 160,000 mg per kg of ash), and bromine, arsenic, and mercury concentrations were moderately high (up to 1500 mg per kg of ash) [27]. Ash samples were also comprised of rare metalloids like bismuth among the other common metals [26]. Soil samples collected in open burning areas also contained dioxin-related compounds (DRCs) including chlorinated, brominated and mixed halogenated dibenzo-*p*-dioxins/dibenzofurans (PBDD/Fs, PCDD/Fs, PXDD/Fs); PBDFs were the most prevalent in samples suggesting that the repetitive combustion of plastics is the main source of chemical contamination [28]. Another study linked the burning of metallic compounds containing copper, lead, zinc, and bromine to the formation of DRCs using soil samples taken from burning areas in Agbogbloshie [29]. The electron transfer from intermediates of PBDE to copper metal may be responsible for a chief portion of the formation of brominated furans at burning sites. Not only is burning metal-rich e-waste conducive for metals to enter the environment, but the chemical processes that take place during burning also provide alternative routes for DRCs to proliferate.

High concentrations of lead and cadmium have been found within dust samples near the community church and e-waste weighing stations [25]. These metal concentrations that originate from e-waste burning were also found in places where livestock and local urban fauna reside and graze. For many people residing in Old Fadama or in close proximity to Agbogbloshie, their livestock intended for consumption are exposed to these very same conditions.

### 4.2. Marine Health

E-waste activities have affected Accra’s Korle Lagoon, Odaw River, and coastal waters in startling ways. The Odaw River, which runs adjacent to some of Agbogbloshie’s primary recycling sites, was observed to have high concentrations of copper, cadmium, lead, iron, chromium, and nickel that were displaced from burning and dumping [30]. This river feeds into the Korle Lagoon, which is the main outlet for Accra’s drainage networks, and ultimately into coastal waters of the Gulf of Guinea. Untreated e-waste ends up in Accra’s waters through dumping, and contaminants in surface dust are easily washed into waterways during floods due to the area’s low-lying topography [31]. Alarming marine consequences have been observed as early as 2002 [32,33] including smaller, sicker, and sparser fish stocks. E-waste activities adversely affect aquatic plant and animal species of all forms, while indirectly affecting humans via consumption of fish and seafood that are dietary staples for coastal residents of Ghana.

Significantly higher concentrations of PCBs have been measured from beach samples near the Korle Lagoon relative to other Ghanaian beachfronts [34]. In addition, concentrations found downstream from the scrap processing yard were higher than those found downstream from the CBD, and there is burgeoning evidence that e-waste activities are responsible for the pollution of the city’s coast [34]. The health of aquatic life is also at risk. Most heavy metals and organic pollutants found in the freshwaters and saltwater coasts are detrimental to the behavior, physiology, metabolism, reproduction, development, and growth of several aquatic specimens [35]. Twenty-eight PAHs, 15 oxygenated PAHs, and 11 trace metals and metalloids have been recorded in muscle and gill tissues of demersal fishes in the Gulf of Guinea [33]. The study found that informal dumping has indirectly altered the development tracks of numerous species, and the practice allegedly increases the risk of cancer in humans via fish consumption.

## 5. The E-Waste Management System

Every day informal workers in Agbogbloshie transform used electronic products into working units and valuable scraps of metal for reuse in secondhand formal and informal markets. International regulatory frameworks (especially the Basel and Bamako Conventions) are supposed to oversee the Ghanaian system, but non-compliance with existing regulations is the norm. Thus far, the Ghanaian government has done little to manage the informality of this trade. Meanwhile, other African nations also share the blame for Accra’s growing e-waste piles because a substantial portion of shipments that end up in Accra are rerouted from various African ports [6]. 

Due to the murkiness of e-waste economies, as well as the fluctuating value of recycled metals or e-scrap metals (based on local as well as international scrap prices), those atop the hierarchy in the e-waste economy opportunistically set metal prices and reap a disproportionate share of the financial returns. This creates an exploitative environment for those at the bottom of the e-waste economy. In general, the lower the position in this hierarchy, the more dangerous the work: burning plastics, rubbers, metals, and other types of electronic components is commonplace. In the absence of formal regulation and instruction from the Ghanaian government, few health and environmental precautions have been implemented.

### 5.1. International Oversight

E-waste operates with limited global stewardship. Unlike the e-waste streams found in the Global North, e-waste arriving in Accra (and in the majority of the Global South) are generally sourced internationally [36]. That said, the information technology revolution is changing established patterns: China and India are emerging as both sources and destinations for e-waste. The Basel Convention, which went into effect in 1992, outlines international regulations for the trade of hazardous wastes in order to minimize exploitation effects from the waste trade that are felt by non-Annex VII nations like Ghana [37]. Within the Convention’s treaty are policies on the restriction of transboundary movements of hazardous wastes except where it is perceived to be in accordance with the principles of sufficient management [38]. This legislation leaves open a wide window for misinterpretation and loopholes as evidenced by the significant streams of hazardous e-waste originating from nations who have signed this treaty (mainly in the European Union) that still end up in Ghana.

The U.S. remains the only industrialized nation not to ratify the convention’s laws, so the USEPA is the U.S.’ only exporter-end source of accountability for e-waste streams destined for Africa and other regions. USEPA’s policies for the regulation of U.S.’ used-electronic exports are hardly sufficient, thus shifting major responsibility and oversight to the Customs Division in Ghana and other importing countries. For instance, the USEPA only regulates devices containing cathode ray tubes typically found inside of old computer monitors and televisions. Non-cathode ray tube devices exit the U.S. without much regulation; even still, non-compliance by companies and lax enforcement by government officials allow devices with cathode ray tubes to be exported. A U.S. Government Accountability Office (GAO) [39] report noted some 47 U.S. brokers advertising their services to ship American e-waste to Ghana and other destinations. Moreover, web surveillance conducted by the U.S. government observed numerous enquiries about exporting American LCD screens to Ghana, thus countervailing all laws [40]. To begin to remedy the situation, the USEPA in 2010 commenced a collaborative relationship with the United Nations initiative Solving the E-Waste Problem (StEP) in order to improve the tracking of global flows of e-waste via more advanced e-waste management systems [41]. More recently, the USEPA is working on improving its sustainable electronics management efforts by developing domestic recycling practices and reducing the amount of harmful toxins within shipments that leave the U.S. [42].

The African region enacted an e-waste regulatory framework in 1998 under the Bamako Convention. Like the Basel Convention, this treaty aims to reduce the exploitative effects experienced by lower-income African e-waste destination hubs. Twenty-five African states have signed the convention’s treaty [43], but little implementation has taken place to date. The language of the Bamako Convention, while Africa-specific, reiterates and improves upon the preventative measures found in the Basel Convention. For instance, the Basel Convention contains policies on banning the transboundary movements of hazardous wastes to developing nations that lack proper e-waste management systems, but the Bamako Convention’s treaty builds on this by prohibiting the import of all hazardous and radioactive wastes into the African states that sign and ratify it. The purpose of this treaty is to minimize and control the flows of e-waste that travel within and between African states, and to ensure that electronics are disposed of and recycled in an environmentally sound manner [43]. Unfortunately, illegal imports into Ghana (and elsewhere in Africa) have not subsided due to weak treaty enforcement. Even delayed enforcement provides ample time for purposeful mislabeling and other measures to avert future efforts by Ghana Revenue Authority’s Customs Division to enforce the Bamako Convention.

Typically, e-waste traverses international boundaries and evades international surveillance due to wording and labeling tactics. Calling a shipment of end-of-life electronic goods a “charitable donation” suffices for a shipment purpose [44], while exporters retain a profit from the “donation”. Mislabeling has also been uncovered on numerous occasions in Ghana [6]. The share of working electronic goods found inside a typical e-waste shipment generally is about 25% [45]. As a result, the amount of unusable computers, cell phones, appliances, and all other forms of e-waste entering Ghana grows annually, and the amount of e-waste in the global trade is expected to rise by about 8 million tons to 50 million tons in 2018 [46].

Although international oversight has thus far been insufficient for safeguarding workers’ interests, certification schemes developed by international agencies show promise for shaping e-waste governance. The two competing certification schemes that have gained prominence are Responsible Recyclers (R2), developed by the US EPA between 2006 and 2008, and e-Stewards, created by several multinational e-waste NGOs (including the Basel Action Network) after leaving talks regarding R2. Perhaps the most important difference between R2 and e-Stewards—and the reason for their schism—is that the former permits exports of waste that are “legal,” while the latter prohibits it completely in order to conform with the international directive put forth by the Basel Convention [47]. Both certification schemes allow recyclers to use eco-labels to advertise fair trade deeds, and they offer certifications to recyclers who implement e-waste worker “best practices” [48]. Generally speaking, certification schemes do little to ameliorate the bigger issue of growing consumerism, but they provide incremental steps toward formalization and societal change via social pressures and public awakening in the Global South to the negative externalities of unchecked neoliberalism.

### 5.2. Ghanaian Legislation

While Ghana is a signatory of the Basel and Bamako conventions, the country has historically lacked a national e-waste policy framework that would help to reaffirm and better enforce international legislation. The Ghanaian EPA has a wide array of environmental legislations in place, but generally lacks policies specifically related to e-waste [49]. E-waste recycling in Ghana incorporates elements from both the formal sector (upstream processing and end-product distribution) and informal sector (e-waste collection, disassembly, and segregation) [50]. Negative human and environmental impacts that are a result of informal recycling are a direct consequence of the lack of Ghanaian government intervention. The government has attempted to intervene on a small scale by implementing workshops and informational seminars to increase awareness regarding the dangers of handling toxic materials [49]. Nevertheless, local knowledge of human health risks remains low due to the voluntary nature of the workshops and the ad-hoc and piecemeal way that e-waste management evolves without a comprehensive strategic framework.

In 2012, a bill known as “The Hazardous and Electronic Waste Control and Management Bill” was introduced to better control and manage hazardous waste that enters Ghana [51]. The bill proposed a tax on imports that was specifically intended to finance more environmentally sound and formal recycling practices, such as the phasing-out of equipment containing PCBs by 2025. In order to reduce the amount of damaged and unusable electronics that pile up on and in the ground in Ghana, manufacturers and distributors would be required to take back the discarded goods that are intermingled within shipments. An “Electrical and Electronic Waste Management Fund” would also be supported by government funds and finance e-waste-related research and reports as well as fund public education awareness about e-waste and recycling. A Board of Trustees comprised of representatives from seven different Ghanaian government agencies would manage this collective fund [52]. This bill—the first e-waste specific legislation considered by the Ghanaian Parliament—was finally ratified in July 2016, and bodes well for Ghana’s future ability to fulfill international obligations and mitigate local e-waste externalities, but with some caveats. Forcing exporters to take-back shipments may be impossible to enforce due to fictitious naming of entities. This bill is also complicated by the reality that these items would be classified as “hazardous” under the Basel Convention and thus illegal to re-export into many countries.

### 5.3. Flows of E-Waste through Accra

Shipments of used electronic goods enter Ghana largely through other African ports. The majority of the e-waste that ends up in Agbogbloshie first enters the continent through South Africa via Durban, Tunisia via Bizerte, and Nigeria via Lagos [6]. These are three of Africa’s most active ports, so shipments containing hazardous materials circumvent the Basel and Bamako Conventions by passing through hectic environments [6]. For shipments sent directly to Ghana, the port city of Tema serves as another major entry point for e-waste [44]. Currently there is little government enforcement of checking devices for reparability, so exporters are not held accountable for the quality of their goods. Of the 280,000 metric tons of e-waste that entered Ghana in 2009, only 1% of shipments were processed through a formal facility [52]. 

Once in Ghana, a shipment will more likely reach an informal facility in Accra where end-of-life electronic goods, including high scrap-value goods like automobiles, will pile up in one of several locations in the city. Scrap collectors pick out devices for dismantling or refurbishing, and bring them to one of several informal facilities within the Agbogbloshie market. A field reconnaissance study performed in 2012 observed approximately 30 repair facilities, with most specializing in a particular type of e-waste. While specializations were evident, some informal facilities were unspecialized, containing various recycled components from cell phones to automobile parts [6]. 

The e-waste hierarchy and distribution of work and income are presented in Table 1. Individuals that partake in this informal system generally lack education, adults and children alike [31]. While workers are cognizant of the fact that higher education leads to occupations with greater pay and lesser workloads, the allure of a starting wage that is upwards of five to seven times that of the monthly wages of a Ghanaian minimum wage employee [53] is often too attractive to resist. Importantly, e-waste is a quick cash business and money changes hands immediately, so workers generally do not wait long to get paid.

The e-waste that has been either refurbished/repaired or recycled for extracted heavy and precious metals will later arrive in the hands of a middleman. These e-waste intermediaries may be scrap collectors who have ascended within the industry due to their connections in Accra and with international players and/or their monetary power [6]. Many scrap dealers are connected to international scrap firms that are located in the Tema Export Processing Zone. These entities send Ghanaian recovered copper, mixed scraps and other metals to international recycling firms in Europe, China, India, and the Middle East. These international players have greater technical capabilities and accumulate scraps from numerous e-waste hubs, thereby achieving economies of scale in recycling [13]. The level of specialization within the e-waste economy means that some degree of job mobility is possible for a select few. There are well-defined roles for middlemen who in turn negotiate with scrap dealers, and scrap dealers in turn interface with export processing zone (EPZ) firms and scrap importers outside of Ghana. At the same time, some streams of refurbished and repaired consumer goods, as well as extracted metals, re-enter the Ghanaian domestic market, where firms in Ghana secure locally processed copper and aluminum fractions from scrap dealers. The non-valuable fractions and the unusable/irreparable e-waste components either end up in a formal landfill or in an informal dumping ground to be burned [13,54].

International non-governmental organizations (NGOs) are beginning to fund and implement new pilot projects aiming to increase formal e-waste recycling. Pure Earth (with funding from the United Nations Industrial Development Organization) and the Global Alliance for Health and Pollution opened an e-waste recycling center in 2015 with automated wire stripping units. The Ghanaian government and municipal authorities are chronically challenged to scale up pilot projects due to the lack the financial and technical capacities to implement integrated waste programs at national and urban scales [50]. Project buys-in is often difficult to secure from informal workers both due to their collective camaraderie, and the reality that many temporary or part-time workers resist an organizational structure that requires a long-term or full-time commitment [50]. Opportunities in formal recycling will also be more limited for informal workers. A take-back scheme implemented by the Dutch company Fairphone and its Ghanaian partner Recell has collected over 60,000 end-of-life mobile phones from e-waste sites by paying a premium for each unit. However, this pilot scheme has also encountered institutional obstacles. As of early 2016, the parties have failed to secure a permit to export end-of-life devices for safe dismantling in Belgium due to the toxic designation of non-working devices. Despite being motivated by principles of environmental ethics and fair labor, both of these initiatives are still non-governmental attempts to organize the Ghanaian e-waste economy using Western models.

### 5.4. Rudimentary Recycling Practices

After manually dismantling electronic devices, recyclers (see Table 1 for economic roles) will normally burn computer components in order to extract metals from plastic, metallic, and rubber coverings. Copper is a common metal for a recycler to specialize in as it is moderately abundant in most electronic goods and fetches roughly US$3.91 per kg in the Agbogbloshie market [6]. Copper wires are burned in order to melt away their rubber coatings and make them presentable for selling [55]. Another common technique for extracting metals is to burn old foam on top of consoles to melt away plastic casings to facilitate the extraction of copper and iron remnants [2]. 

Gold, more of a trace element in most electronics, is found inside of the printed circuit boards of computers in roughly 0.26 g quantities [6]. High gold prices have driven interest in gold reclamation from e-waste. Informal recyclers in Ghana do not currently have the technical capacity to extract gold, so scraps are grinded down into a fine-grain powder and exported to nations with sophisticated machinery that can extract gold and other rare metals. Burning releases harmful toxins including lead, tin, and hydroxylated PBDEs [56,57]; the grinding of motherboards only exacerbates this pollution. 

As already noted, burning remains the main medium through which toxins enter the air and tissue of people and animals in Agbogbloshie. Indiscriminate dumping of e-waste into open fields and bodies of water is a major contributor of environmental toxication as well. The land cover of Agbogbloshie and its surrounding areas is frequently riddled with different types of unusable electronic parts, most of which are ultimately burned and crushed. Around Agbogbloshie, cattle, goats, and other livestock, which are still raised for meat consumption, are seen grazing on the dust-laden spaces, thus inadvertently consuming e-waste toxins [3]. Due to the growing accumulations of e-waste and the lack of proper disposal sites, e-waste residues also enter the nearby Korle Lagoon and Odaw River [55], leading to the further contamination of the Lagoon that feeds into Accra’s coastal waters. 

## 6. The E-Waste Worker Experience 

The voices of e-waste workers remain largely absent throughout the e-waste literature. Understanding the lived experiences of workers is of great importance because such testimonies help create more holistic images of life on the ground. Photographs of young men leaning over dark smoke clouds surrounded by rubbish and ash have permeated international public spheres; while such images often bring public attention to the adverse health outcomes and living environments of these workers, they often obscure the benefits workers receive from their work. For e-waste workers, collecting scrap and burning metals over open flames are sometimes deeply rooted in building their identity, pride, and better futures [58]. Informal workers, like workers in all professional fields, are producers of knowledge and reality [59]; if such realities continue to be framed by hysteria, then social equality, understanding, and in turn, inclusion will be difficult to attain. Therefore, policy makers must successfully interlace the perceptions and understandings of those physically partaking in work for e-waste policy to follow a sustainable trajectory.

The consequences of the e-waste trade on the quality of life for those participating in and living in proximity to the Agbogbloshie recycling hub are particularly overlooked largely due to media sensationalism. Despite a large and growing body of research on human and environmental exposure to e-waste, the voices of the workers that participate in the e-waste regime are frequently muted and neglected. Without understanding the lived experiences of those involved, we cannot fully appreciate the challenges in Agbogbloshie, and the accumulation of practical knowledge about recycling remains incomplete. Exploitation is apparent, but the perceptions of workers from all strata within the e-waste space economy are under-examined. E-waste management thus should be inclusive and partner with informal economic actors instead of being imposed from the top-down [50]. Two particular social dimensions that have received some scholarly attention in Accra are the health perceptions and job satisfaction of e-waste workers. 

### 6.1. Workers’ Health Perceptions

When asked about the risks associated with the e-waste processing, Agbogbloshie workers’ environmental and human health perceptions are not aligned with the findings of numerous risk assessment studies. In general, workers fall within a continuum ranging from softened ideas of the attendant risks to unawareness [60]. Interviews with workers revealed that almost half of e-waste workers in Agbogbloshie were more concerned with everyday hazards like burns and occupational injuries than potentially worse future side-effects such as respiratory problems and cancer [60]. 

The lack of affordable healthcare for informal workers limits visits to doctors and hospitals, so expert medical opinion on e-waste related health implications is limited [61]. Most e-waste workers rely on self-treatment and traditional medical practitioners that disburse traditional medications, herbal preparations, and chemical treatments [61]. The vast majority of e-waste workers in Accra do not use protective measures like masks, gloves, goggles, or medications, and approximately half of workers did not believe that they had the ability to prevent e-waste-related hazards [14]. The lack of preventative measures used by e-waste workers thus seems to be associated with low health awareness, limited sense of agency in protecting themselves, limited financial means, and lack of public education. Workers’ tolerance of workplace risks provides further evidence of the lack of decent work in Accra’s informal economy and thus limited opportunities for switching occupations. 

### 6.2. Job Satisfaction

In a survey of e-waste workers, participants reported feeling unhappy with their working conditions [54]. The e-waste trade for most of the interviewed workers served as a means to earn and remit money back to their families, most of whom still lived in Northern Ghana. Quick cash from e-waste jobs continues to draw informal workers into dangerous employment conditions.

It is estimated that the turnover rate of employment for e-waste workers is between three and seven years [54]. During their time working in the e-waste business, several interviewees reported headaches, nausea, insomnia, and respiratory problems among other symptoms, but continued to work out of necessity [3]. In essence, the few years working in the e-waste trade are laborious and full of discomfort, but the rewarding financial compensation trumps most feelings of self-pity especially when alternative employment opportunities are virtually non-existent.

## 7. Policy Considerations 

Academics, international organizations, and NGOs have proposed many different mitigation techniques that could potentially produce a more ethical and fairer e-waste economy. Most policy frameworks coalesce around two notions: (1) improving producer responsibility in e-waste origin nations; and (2) formalization of the industry in e-waste destination countries. Built-in obsolescence is a growing concern as well: electronics are made to last for a short amount of time to pave the way for new generations of the same commodity that in turn rely on further supplies of finite resources. Efforts to re-engineer electronics with an emphasis on dismantling and recycling valuable metals are emerging (e.g., the Fairphone), but for the most part consumer aesthetics and corporate profits remain the driving force.

### 7.1. Extended Producer Responsibility

Electronics producers lack accountability in solving the e-waste predicament. They operate without regulations that govern the types of materials used in their products. The Extended Producer Responsibility (EPR) approach aims to shift the responsibility for contaminants found in e-waste from municipalities in the developing world—and specifically neighborhoods like Agbogbloshie—to the producers, i.e., companies such as Apple, Toshiba, and Samsung [62]. Under the EPR umbrella, take-back programs, leasing programs, and deposit-refund schemes are gaining popularity, and, in some cases, they prohibit the incorporation of certain hazardous materials inside devices [63]. This approach aims to keep hazardous materials in check by enhancing producer responsibility and ultimately reduce the quantities that stockpile in e-waste destination nations. However, EPR in practice has been token rather than transformational, and efforts to date have lacked serious financial backing.

From a contemporary business lens, there is insufficient financial incentive for EPR to become a global norm; this may change as class action legal suits against global digital companies and others emerge on behalf of claimants whose lives have been adversely affected by poor health linked to e-waste. In terms of producer responsibility, the European Union’s (EU) Restriction of Hazardous Substances (RoHS) Directive that was introduced in 2003 sets the framework for EPR in member countries. Accordingly, products created in EU countries may not include metals like lead, mercury, and cadmium, or flame retardants like PBDEs and PCBs, specifically to reduce the amount of toxins in consumer circulation over time. However, the effect of the RoHS Directive has been limited because most consumer electronics products are made in China rather than the EU. The legislation also provides for collection schemes so that corporations can recycle their obsolete electronics properly [64]. Again, since digital devices are mainly produced outside of the EU, the RoHS Directive only provides a framework for a minor portion of used electronics. Meanwhile, Ghana is participating in a Swiss environmental initiative called “The Sustainable Recycling Industries” (SRI). This partnership involves technology collaborations and business opportunities in EPR to promote longer-term functioning of recycling businesses [52]. The SRI initiative began implementing its ideas for on-the-ground activities in June 2015 [65], but as of early 2016, EPR plans were still in the works and had not yet been implemented.

### 7.2. Formalization

To call the areas where e-waste is recycled and processed in Agbogbloshie “facilities” is somewhat of a misnomer. The word “facilities” implies a high level of physical infrastructure as well as a formal managerial presence with some degree of legal accountability. Operations in Agbogbloshie are seriously lacking in both these domains; the absence of physical facilities and sophisticated technologies throughout the e-waste business in Ghana has been detrimental to human and physical environments.

Talk of formalization prevails in the e-waste literature [66]. Formalizing the e-waste extends well-beyond creating designated recycling areas in contained buildings; this process would also entail increased border control and regulation, and some organizations (e.g., StEP) go as far as to advocate for the positive incorporation of e-waste workers into the formal economy. In practice, e-waste imports could be thoroughly examined, and individual objects could be assigned tracking numbers so that their recycling statuses could be monitored throughout the major processes of transformation. However, could all processes and transformations be tracked? Contemporary smart phones incorporate 40+ metals and rare earth elements. Which metals would be the focus and where? To incorporate e-waste workers into the Ghanaian formal economy, the government could consider hiring those who have created positions for themselves in the e-waste trade, as they have firsthand experience with recycling. This formalization process could potentially shortchange many current e-waste workers, as there would be no guarantee that they would receive a formal position. However, as far as health is concerned, a formal employment regime would provide employees with appropriate safety measures. One example that has been proposed for Ghana is the Best of Two Worlds (Bo2W) Project (originally implemented in Bangalore, India), which aims to create environmentally sound collection and recycling practices. Proponents of the Bo2W model aim to link local recyclers with international markets, improve metal extraction techniques, and monitor all activities [52]. The predecessor of Bo2W, the “Clean E-Waste Channel” model, rolled out in Bangalore in 2008 and was hailed as the first successful mutually beneficial scheme for recyclers and corporations. Within informal worker groups, however, this initiative proved to be troubling as it puts e-waste in the hands of fewer recyclers [67]. For future “win-win” implementation schemes, these models must be revised to allow more individual recyclers to participate in the process rather than necessarily forcing consolidation.

A closely monitored and regularized e-waste management regime would both contribute to economic growth as well as help alleviate extreme poverty [68]. Nearly 70% of Ghanaians create jobs for themselves in the informal economy; informal work in e-waste provide employment opportunities to struggling citizens facing harsh economic realities [68], but e-waste formalization presents an opportunity to bolster livelihoods. Traditionally, waste picking and recycling were heavily stigmatized as occupations of the most marginalized members of society. However, a more contemporary lens of sustainability could explicitly link cadres of informal faceless pickers engaged in dirty work with global, branded green recycling companies. This approach could modernize outdated perceptions about employment roles within the waste economy, thereby bringing e-waste into the mainstream and promoting a greener future. This shift could be rapid if government recognized and embraced the informal economy’s potential in e-waste reform legislation [5].

If the health dangers associated with the e-waste trade could somehow be stripped away from businesses through improved monitoring systems and increased governmental regulation, perhaps formalizing the entire e-waste regime, as many have called for, may not be necessary [68]. Connecting the informal and formal economies in time and space [69] would allow the entrepreneurial and innovative essence of the trade to survive, within the context of moving on a sustainability trajectory. Above all else, symbiotic relationships between actors within the e-waste economy could be developed [70], and this would go a long way toward acknowledging that informality is a norm rather an exception in the rapidly urbanizing Global South.

## 8. Conclusions 

After roughly a decade of e-waste research in Accra, where are we now? Agbogbloshie’s landscape was transformed profoundly through Ghana’s entry into the digital revolution. This informal settlement is adversely incorporated into the international e-waste economy. Most studies have focused on the visible transformation above ground, but we have synthesized the less-visible and chemical transformations of human bodies, soils, waters, air, and to some extent food chains and the evolution of an e-waste hazard area. Even if the area could return to its original purpose (which is extremely unlikely and problematic without community buy-in), it would be difficult to estimate how long the toxins would remain. No assessment of the medium and long-term damages on the health of human and natural environments exists. 

This burgeoning corpus of e-waste research in Accra invites conversation as well as caution about Agbogbloshie’s global notoriety as an apocalyptic landscape on the edge of the digital technology revolution. This representation may make headlines, but it is also dangerous and invokes knee-jerk national and municipal responses of demolition and dismantling that are uninformed by the extensive scholarship on e-waste and informality in Agbogbloshie. Without a comprehensive understanding of the space economies of e-waste, demolition may simply shift and diffuse the locus of activities to multiple sites. This might spread and magnify e-waste’s negative externalities while creating greater metabolic challenges for urban environments. 

Given the complexity of e-waste circuitry that links very different actors into the e-waste space economy from large formal recycling firms like Umicore (Belgium) to informal part-time workers in Agbogbloshie, the status quo continues to prevail. Governments and consumers continue to focus on other issues, and those involved in the e-waste circuitry lack incentives to change existing practices. Without global and local integrated management of e-waste, the burden on Accra and other sites in the Global South will continue to spiral. For instance, e-waste inflows into Ghana are projected to double by 2020 [12], and Ghana’s own growing e-waste stream (i.e., as a producer of e-waste) is anticipated to add a new dimension.

The e-waste literature is unequivocal on how multiple actors and institutions exploit human and environmental health in Agbogbloshie, even after a decade of research findings. A few adaptation strategies have been implemented (e.g., bans on burning, and pilot projects that use mechanical shredding and buy-back schemes to improve livelihoods and explicitly link global and local recycling efforts), but they have hardly made a dent in changing existing practices and in modifying the business-as-usual approach. The impacts of various pilot recycling schemes need to be assessed, and their potential for scaling up and/or replacement by alternative projects needs more attention. Surprisingly, mitigation strategies have been neglected. Local livelihood enhancements need to be incorporated into any e-waste solution so that e-waste management has a chance to move toward a sustainable trajectory [68]. This will not be easy, as the prevailing power symmetries in e-waste economies will need to be modified. However, our close read of the literature informs us that engagement of distant and hitherto separated economic actors—formal recycling firms, informal waste pickers and processors, scrap metal traders, and especially the communities where e-waste is recovered—is now a real possibility especially in light of the United Nations’ Sustainable Development Goals for 2015–2030.

The prospect of future bulldozing exercises in the Agbogbloshie and Old Fadama communities threatens the status quo centralization of e-waste commerce as well as opportunities for formalization of recycling activities, particularly related to burning and dismantling. Bulldozing would likely push these activities out of central Accra and germinate many decentralized pockets of e-waste activity beyond Accra—a trend that has anecdotally already begun as recyclers experiment with performing some dismantling steps at new break-of-bulk points in peri-urban Accra. These new sites remain largely in the shadows to scholars and regulators by design. Future bulldozing thus may accelerate the quietly shifting geographies of e-waste, complicating the crafting and implementation of new management policies. A sustainable trajectory requires policy makers in Accra to consider and embrace informal workers in Agbogbloshie as integral members of the greater Accra socioeconomic system and to reconcile everyone’s best interests rather than fracturing and disrupting existing e-waste networks and livelihoods. 

A decade of research has made visible the circuitry in e-waste that traverses multiple borders and links otherwise unconnected places and peoples. Any solution to e-waste will have to think through how the valuable fractions of processed scrap exports such as copper, gold, and rare earth metals can subsidize the sound recycling of non-valuable fractions such as plastics, LED screens, and other residuals. Tacit acknowledgment of the linkages between informal e-waste practices and formal global businesses may be the key to tackling the present e-waste challenge and its local manifestations. Uncovering the occluded functioning as well as the real impacts of e-waste on human lives, health, and communities has been the major contribution of e-waste research to date. Moving scholarship toward devising and assessing more sustainable solutions that are participatory and fair to low-income workers presents one of our greatest challenges of the next decade.

## Figures and Tables

**Table 1:** Hierarchical structure of e-waste labor segments, with worker incomes reported from Agbogbloshie.

Role in E-Waste Circuitry	Estimated Average Monthly Income (USD) *	Percent of Ghanaian Daily Minimum Wage *	Main E-Waste Activities
Global Firm	N/A	N/A	Global recycling company that imports raw and processed scraps from abroad and from within Ghana. Performs more sophisticated refining of materials and exports products to global clients.
International Firm	$20,000+	N/A	Formal recycling export firm located in export processing zone in Tema. Buys from scrap dealers and finances own scrap collectors.
Scrap Dealer	$1500	2747%	Commands e-waste operations at the top of the e-waste trade hierarchy; negotiates deals with players in the formal economy.
Middleman	$1050	1923%	Acts as an intermediary between scrap dealers and recyclers. Well-connected individuals who prepare recycled scraps for resale.
Refurbisher	$190–$250	348%–458%	Repairs non-functioning electronic goods to be sold in the Ghanaian secondhand electronics market.
Recycler	$175–$285	321%–522%	Picks through e-waste in order to extract metals. Individuals may or may not choose to specialize in a particular type of electronic. Many recyclers partake in open burning as an extraction technique.
Scrap Collector	$70–$140	128%–256%	Collects obsolete electronics in carts or rentable cars-for-hire. Collectors either buy e-waste from consumers or scavenge for parts at dump sites.
Child Laborer	≤$20	≤36.6%	Males participate in recycling or collecting. Young girls may be found distributing water for consumption and fire control.

* Income and wage figures are calculated using the March 2016 exchange rate of 1 USD = 0.26 GHC.
